# Ankylostomiasis

**Published:** 1905-09-09

**Authors:** 


### ANKYLOSTOMIASIS.

It is well known that the disease variously spoken of as Egyptian chlorosis, brick-makers' anæmia, miners' anæmia, and mountain anæmia depends upon an infection of the intestinal tract by the nematode commonly called the Ankylostomum duodenale. The organism generally enters the system by the mouth, and affixes itself to the mucous membrane of the duodenal region of the intestinal canal. But it is important to know that infection may take place through the unbroken skin. This appears to have been experimentally achieved by Dr. A. E. Boycott.[1] Having secured a willing victim in the courageous person of Dr. J. B. Leathes, Dr. Boycott procured some encapsulated larvæ, artificially hatched from eggs derived from infected fæces in Cornwall. These larvæ were applied, in water, to the forearm of Dr. Leathes and left for five minutes. The arm was then bandaged for 24 hours, after which interval the bandages were removed and the arm thoroughly cleansed. Symptoms of infection, slight, though definite, were observed on the day following the experiment, in the shape of roundish patches of erythema, which showed no progress and disappeared in a few days. The site of the application began to itch on the same day and remained irritable for a matter of two weeks. There was a very mild degree of dyspepsia and also of bronchial catarrh, neither of which was habitual to the subject of the experiment. The eosinophilia characteristic of the infection became apparent on the twenty-seventh day following the inoculation, and on the fiftieth day the ova of the worm were discovered in the stools. On the fifty-third day 40 grains of thymol were administered, but ova were again recovered from the stools on the sixty-second day. A second dose of thymol, reaching 90 grains, given in three doses, resulted in an almost entire disappearance of the ova. The delay in the cure is attributed by Dr. Boycott to the variable speed with which the larvæ reach the intestine.

The eosinophilia was first observed twenty-seven days after infection and twenty-three days before the appearance of ova in the fæces. It rose gradually, and finally reached the high figure of 45 per cent. There was no leucocytosis of any importance, and the curve of the eosinophilia appeared to be practically the same as that observed in cases of mouth-infection.

It seems therefore that the worm begins to produce the substance to which the eosinophile cells react, at a stage of development corresponding to the third week after its entrance into the body. This eosinophilia is very persistent. The author quotes the case of a man who while infected showed a percentage of about seventeen eosinophiles in a differential count of white cells, and who still showed a percentage of eleven, thirty months after the commencement of treatment, and more than twenty months after his restoration to health and the disappearance of ova from his stools.

As to the course of the infection, when the former takes place through the skin, Looss has shown by experiments with the Ankylostomum caninum upon dogs, that the larvæ leave the skin in the blood-stream and pass by this means through the right heart to the lungs; here they are arrested, and go by way of the trachea into the œsophagus and thus to the intestine. These observations have been confirmed by Schaudinn, working with the Ankylostomum duodenale and monkeys.

[1] Jour. of Hygiene, July 1905.

